# Carcinosarcoma of native renal pelvis in recipient after a renal transplant: a case report

**DOI:** 10.1186/1477-7819-12-407

**Published:** 2014-12-30

**Authors:** Jitao Wu, Xuyun Wang, Chunhua Lin, Shengqiang Yu, Li Cai, Zhenli Gao

**Affiliations:** Department of Urology, Yantai Yuhuangding Hospital, No.20 East Yuhuangding Road, Yantai, Shandong Province 264000 China; Department of Pathology, Yantai Yuhuangding Hospital, No.20 East Yuhuangding Road, Yantai, Shandong Province 264000 China

**Keywords:** Carcinosarcoma, Renal pelvis, Renal transplantation, Sirolimus, Pulmonary cytomegalovirus infection

## Abstract

**Background:**

Carcinosarcoma is a malignancy that rarely occurs in the renal pelvis.

**Materials and methods:**

We present a case of histologically proven, native renal pelvis carcinosarcoma in a 65-year-old woman who had accepted a renal transplant. We performed a laparoscopic ureterectomy, combined with lymph node dissection and immunosuppression with sirolimus (SRL), which was alternated with the conventional immunosuppressant - cyclosporine.

**Results:**

This patient was still alive 34 months after renal transplantation.

**Conclusions:**

Operation is still the best choice, and the SRL may be beneficial for preventing the progression of a tumor.

## Background

The overall incidence of *de novo* malignancies in renal transplant (RT) recipients is significantly higher than in the general population due to long-term immunosuppressive therapy and oncogenic viral infections [1, 2]. The malignant tumors of the transplanted kidney are mainly nonmelanoma skin cancers and malignancies, including post-transplant lymphoproliferative disorder and solid organ cancer [3, 4]. The prevalence and characteristics of post-transplant malignancies differ in various geographic areas. In western countries, the most common type of post-transplant urinary tract malignancy is renal cell carcinoma [1]. In contrast, the majority of reported tumors in Chinese patients were urothelial carcinomas [5, 6], which occurred in 4.1% of all RT recipients and 47.6% of all patients with a post-transplant malignancy [6]. Carcinosarcoma of the kidney and renal pelvis (CSKP) commonly presented as high-grade, advanced stage disease, and was associated with a poor prognosis regardless of location [7].

Carcinosarcoma of the renal pelvis is an extremely rare malignancy with a poor prognosis in the few reported cases [8–11]. The carcinosarcoma of the renal pelvis in patients after renal transplantation is rarer. Here, we present a histologically proven case of carcinosarcoma of the left renal pelvis in renal transplant recipient.

## Case presentation

**Figure 1:**
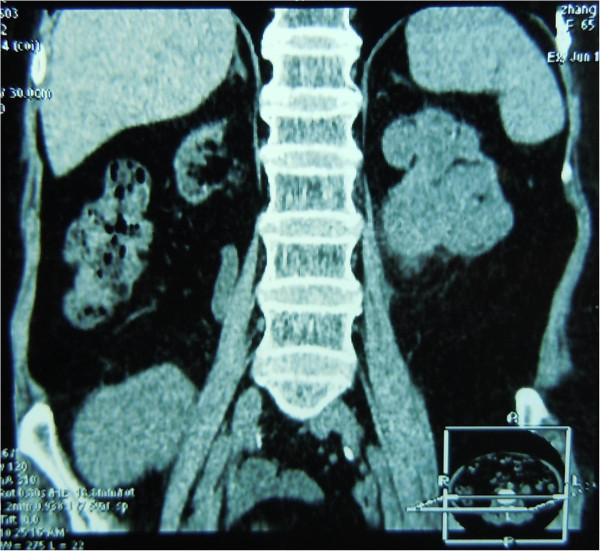
**Computed tomography (CT) scan revealed a 5 cm × 4 cm lesion in the left pelvic region.**

**Figure 2:**
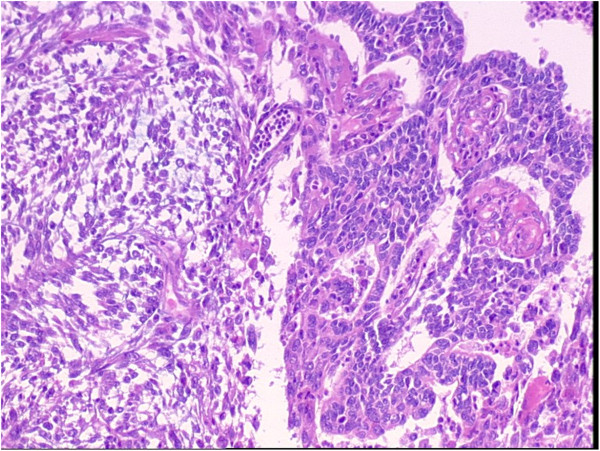
**Pathological examination of the lesion.**

**Figure 3:**
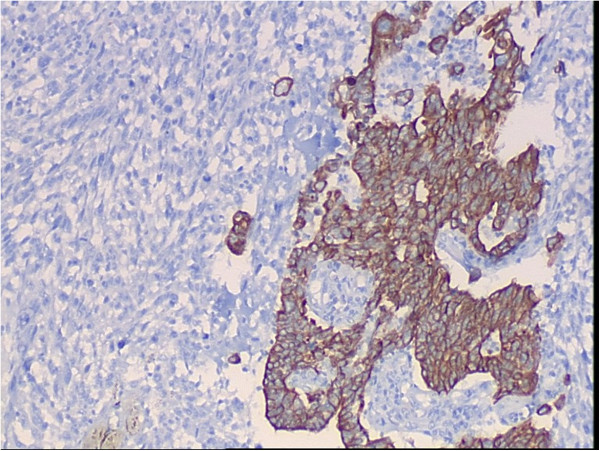
**Immunohistochemical examination of the cytokeratin.**

**Figure 4:**
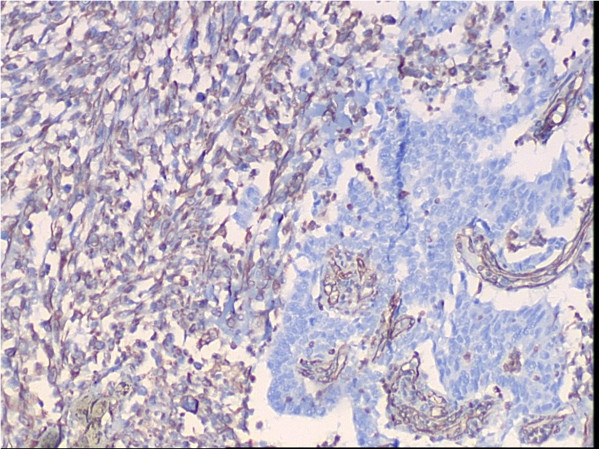
**Immunohistochemical examination of the vimentin.**

**Figure 5:**
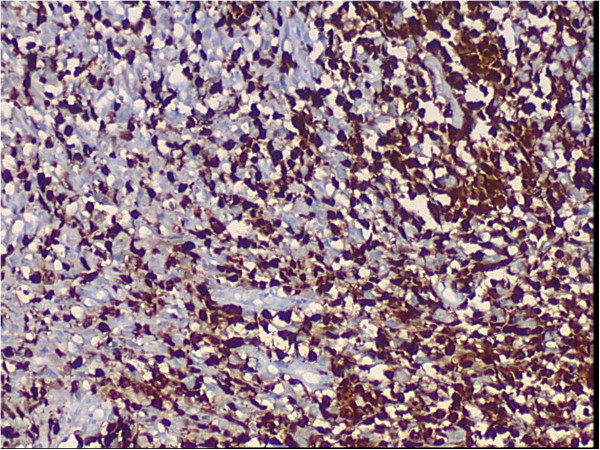
**Immunohistochemical examination of S-100.**

A 65-year-old female patient developed chronic renal failure with unknown etiology and began hemodialysis in 2003. The patient denied past tobacco use or analgesic abuse. The patient received a renal transplantation in 2005 from a male cadaveric donor. Immunosuppressive therapy was applied using a triple immunosuppressive regimen, including cyclosporine (CsA), prednisone, and mycophenolate mofetil.The patient suffered from a pulmonary cytomegalovirus infection 8 months after the renal transplantation, and took oral cyclosporine and prednisone for a combination immunosuppressive treatment after the transplant. She had normal renal function, and the serum creatinine levels were maintained at 0.62 to 0.88 mg/dL. Forty-eight months after the renal transplantation, the patient went to the local hospital because of recurrent gross hematuria with waist pain on the left side. The CT examination revealed a left pelvic lesion of approximately 5 cm × 4 cm (Figure 1). It was showed in the CT image that, irregular nodular soft tissue shadow filled in the left pelvis; the adjacent calyces and renal parenchyma were compressed; and there was no obvious swollen lymph gland shadow. Preoperative cystoscopy and chest computed tomography (CT) examinations revealed no abnormalities.A pathological examination showed that the size of the full-cut kidney and the surrounding fat capsule was 15 cm × 10 cm × 5 cm, and the actual kidney size was 12 cm × 7 cm × 4 cm. A bulging mass of 6 cm × 4 cm was observed at the renal pelvic cut surface without an envelope. This mass showed infiltrative growth that was gray and of fine quality, partly translucent and jelly-like, and also showed some hemorrhagic and necrotic areas. The renal parenchyma was atrophied and the ureteral length was 8 cm. Microscopic observations revealed two tumor components. One was a high-grade urothelial carcinoma component with partial papillary arrangement, and the epithelial cells were mostly heterotypic and pleomorphic, with some of them falling off. The cell nucleus was obviously pleomorphic, deeply stained, and the nucleolus was prominent and visible with pathological mitotic figures. The other was a heterologous sarcomatous component with a malignant peripheral nerve sheath tumor (MPNST) structure, and this was the dominant component. The tumors showed a diffuse growth or formed an alternate distribution of cell-rich areas and cell-sparse areas. The sparse areas were associated with myxoid stroma. The blood vessels were surrounded by tumor cells. The cells were fusiform and polygonal, and the cytoplasm was abundant, with red staining. The nucleus was large, deeply stained and irregular, and the nucleolus was prominent, with visible mitotic figures, multifocal necrosis and hemorrhage. Part of the region showed urothelial carcinoma and MPNST component with a transitional phase (Figure 2). According to the TNM classification system, the patient was diagnosed with stage pT3N0M0.An immunohistochemical examination showed that cytokeratin of the urothelial carcinoma was positive (Figure 3). Vimentin was positive in the sarcoma, and was negative in the urothelial carcinoma (Figure 4). Some of the MPNST cells S-100 were positive in the carcinoma and sarcoma transition area, and strongly positive in the area of the sarcoma cells (Figure 5). Smooth muscle actin (SMA) of sarcoma cells was negative, and Human Melanoma Black (HMB45) and desmin were negative.

The pathological diagnosis was that the left renal pelvic carcinosarcoma invaded the renal parenchyma, but was not involved the ureteral stump.

The patient accepted laparoscopic ureterectomy combined with lymph node dissection in our hospital. The operation went well, and 7 days after the operation, she was discharged from the hospital as she had recuperated. One week after the operation, she began to adjust the immunosuppressive regimen by using sirolimus (SRL) instead of cyclosporine, and the initial loading dose of SRL was 2 mg and 1 mg the next day, which was taken 4 hours after meals. Regular monitoring of blood concentrations was carried out after the conversion, and the drug dosage was adjusted according to the blood concentration, to maintain a plasma concentration of 4 to 6 μg/L of SRL. The patient refused to undergo any adjuvant therapy after the operation. She accepted regular postoperative follow-up. At the time of writing, 34 months after the left kidney ureterectomy, she is still alive with normal renal function.

## Discussion

The overall incidence of *de novo* malignancies after RT is four to five times higher than that in the general population and has been estimated to be as high as 20% after 10 years [1]. According to the Cincinnati Transplant Tumor Registry, the most prevalent *de novo* neoplasms in organ transplant recipients are skin cancers and post-transplant lymphoproliferative disease [1, 2]. For urinary tract, renal cell carcinoma (RCC) was the most frequent tumor after RT in the west [1, 5], with urothelium cancer being most frequent in China [6]. The development of carcinosarcoma is well documented in the mammary gland, the larynx and the uterus. Less frequently, development in bladder, esophageal, lung and kidney has been reported. Fewer than twenty cases of carcinosarcoma of the pelvis have been described in the literature.

Carcinosarcoma is often difficult to distinguish from sarcomatoid carcinoma. Carcinosarcomas are composed of epithelial and sarcomatous components, and sarcomatoid carcinomas are malignant spindle cell neoplasms composed of epithelial components containing changes of sarcomatoid morphology [12]. Microscopically, the carcinomatous component is mostly transitional cell carcinoma and the sarcomatous component is largely composed of spindle or pleomorphic tumor giant-cells. Carcinosarcomas and sarcomatoid carcinomas are similar on hematoxylineosin staining, so immunohistochemical study is mandatory for diagnosis. In this case, epithelial component expressed cytokeratins while sarcomatous component vimentin and S-100. The pathological diagnosis is confirmed.

Exact survival rate remains a matter of controversy due to a lack of consistent long-term follow-up. Wang *et al.* reviewed the carcinosarcoma involving kidney and renal pelvis, and the median cancer specific survival was 6 months and the 1-year cancer specific survival rate for the entire cohort was 30.2% [7]. In the present report, this patient is still alive (>34 months) with normal renal function and no metastasis. This survival may be because regular follow-up allowed for diagnosis and treatment of the tumor in an early stage and because SRL instead of cyclosporine may have suppressed the progression of tumor. Hojo *et al.* has reported that cyclosporine can promote cancer progression by a direct cellular effect that is independent of its effect on the host’s immune cells, and that cyclosporine-induced transforming growth factor-beta (TGF-beta) production is involved in this [13]. That is to say, cyclosporine can also promote cancer progression directly. As is reported, when compared with cyclosporine, SRL induce adverse events more frequently, but the mean MDRD eGFR is higher for renal function [14]. The reintroduction of calcineurin inhibitors is safe in patients who have been withdrawn from sirolimus owing to adverse effects [15]. SRL, besides its antiangiogenic properties, has a strong tumor-specific functional effect on established tumor vessels [16].

Carcinosarcoma is rare in renal pelvis but progresses rapidly and has an unsatisfactory prognosis. The pathologic stage is the main predictor of patient survival. No direct correlation has been found with previous stone disease, infection, or radiation exposure and tumor development. Chemotherapy offers little response and no survival advantage [17–20]. Treatment is early nephrectomy. For this, early diagnosis should be made. Transplanted and native kidneys should be screened for tumors by ultrasound after transplantation [21]. Thus, tumors can be diagnosed at an early stage. RT recipients should do ultrasound and CT in regular postoperative follow-ups to allow for the diagnosis of carcinosarcoma at an early stage. Nonfunctioning native kidneys with suspicious lesions should be removed early [5].

## Conclusion

In conclusion, carcinosarcoma is rare in the renal pelvis, progresses rapidly and has an unsatisfactory prognosis. It is not sensitive to chemo-radiation. Operation is still the best choice, and the SRL may be beneficial for preventing the progression of the tumor. From a therapeutic and prognostic point of view, it is important to make a correct and early diagnosis.

## Consent

Written informed consent was obtained from the patient for publication of this Case report and any accompanying images. A copy of the written consent is available for review by the Editor-in-Chief of this journal.

## Abbreviations

CsA: cyclosporine
CSKP: carcinosarcoma of the kidney and renal pelvis
CT: computed tomography
MPNST: malignant peripheral nerve sheath tumor
RCC: renal cell carcinoma
RT: renal transplant
SRL: sirolimus.
